# First person – Avik Banerjee

**DOI:** 10.1242/bio.062276

**Published:** 2025-10-08

## Abstract

First Person is a series of interviews with the first authors of a selection of papers published in Biology Open, helping researchers promote themselves alongside their papers. Avik Banerjee is first author on ‘ Stress-induced elemental retention in an ectothermic vertebrate’, published in BiO. Avik is a PhD student in the lab of Maria Thaker at the Centre for Ecological Sciences, Indian Institute of Science, Bengaluru, India, investigating how animals make dietary nutritional choices under different ecological contexts and how these choices affect their survival and reproduction.


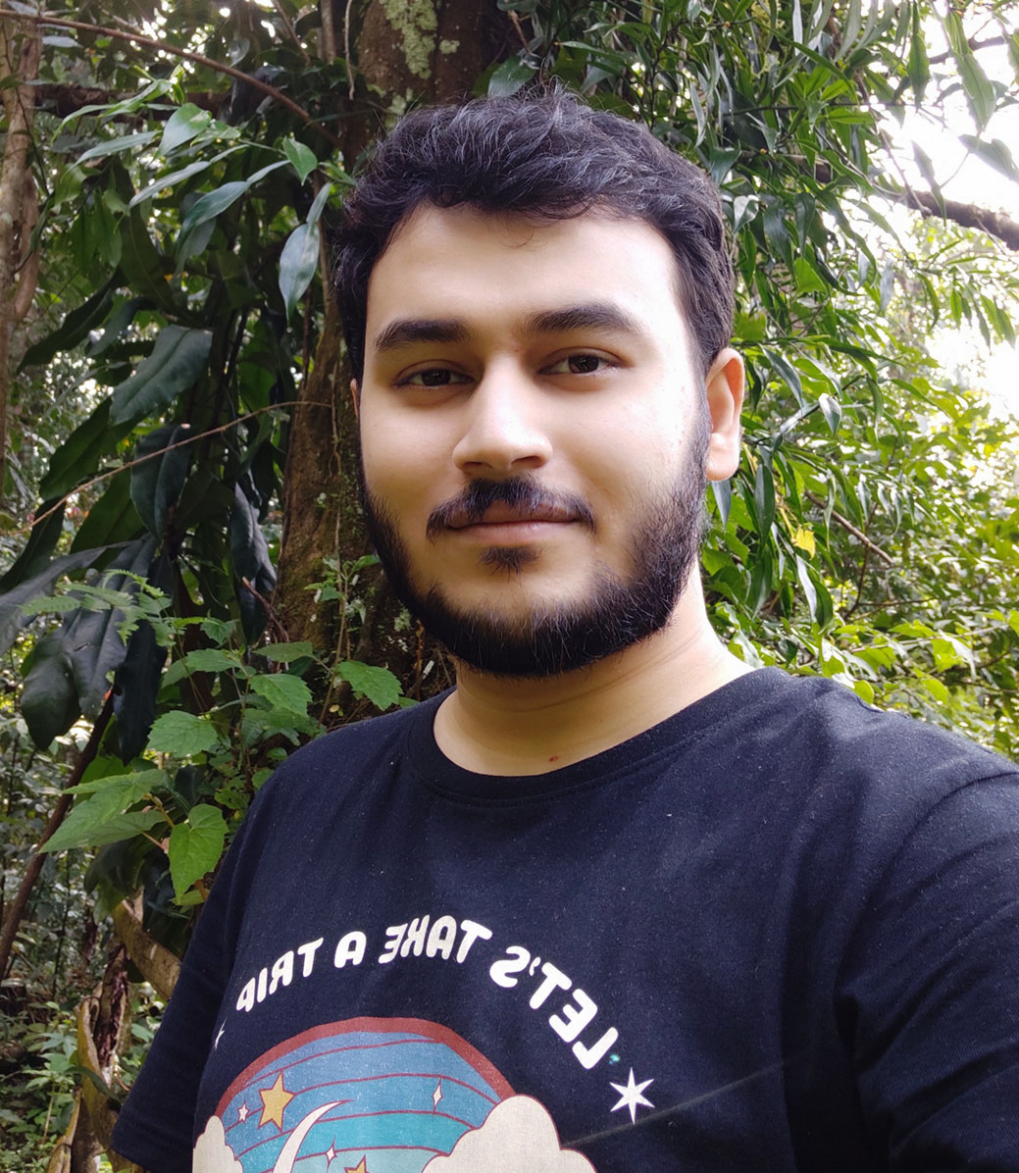


**Avik Banerjee**

**Describe your scientific journey and your current research focus**

I realised that am keen on doing research only during my Master's when I was introduced to the fascinating fields of ecology and animal behaviour. The more I studied these fields, the more I became interested in exploring them. I joined IISc, Bengaluru as a PhD scholar in the macrophysiology lab of Prof. Maria Thaker to study nutritional ecology of lizards. As food contains multiple nutrients that are required in specific ratios depending on an animal's physiological needs, foraging decisions of animals should reflect their nutritional demands under natural ecological conditions. During my PhD, I investigated how different environmental risks such as resource uncertainty, seasonal challenges, and predation risk influence physiology, behaviour, and nutritional demands in the tropical lizard, *Psammophilus dorsalis*. Alone the way, my findings have showed that lizards can adjust their foraging based on shifting physiological needs unravelling novel strategies (for example, risk-sensitive foraging in reptiles) and offering new insights to the field. These results have inspired me to further explore how animals' shifting foraging choices influence their fitness in the long run, a direction I intend to pursue in future work.

**Who or what inspired you to become a scientist?**

I have always found it interesting to observe animals in the wild, yet I never imagined myself pursuing a career as a scientist growing up. During my undergraduate years, as I started reading scientific papers and attended some inspiring lectures on ecology and animal behaviour, I realised how exciting it is to ask my own questions, design experiments, and uncover answers. Along the way, many professors and mentors have inspired me to work hard and keep learning, a pursuit that, to me, captures the essence of being a researcher.

**How would you explain the main finding of your paper?**

We examined whether lizards cope with physiological stress by retaining more nutrients. Stress responses increase energy usage and metabolism, so animals are expected to increase intake of carbon-rich nutrients (such as carbohydrates and lipids which are energy providing macromolecules), or, if food is restricted, hold onto more nutrients from what they have eaten. To test this, we studied lizards that were either placed under stress treatment or kept as controls (undisturbed). Our study found that there was no change in retention of nutrients for both the treatment and control groups.

**What are the potential implications of this finding for your field of research?**

We found that lizards did not rely on nutrient retention to meet the energy costs of stress highlighting the resilience of animals under challenging conditions. To the best of our knowledge, this is the first test of stress-induced elemental retention in a vertebrate, and it highlights the need for more research across taxa to understand how animals regulate nutrients in response to stress under diverse ecological conditions.

**Figure:**
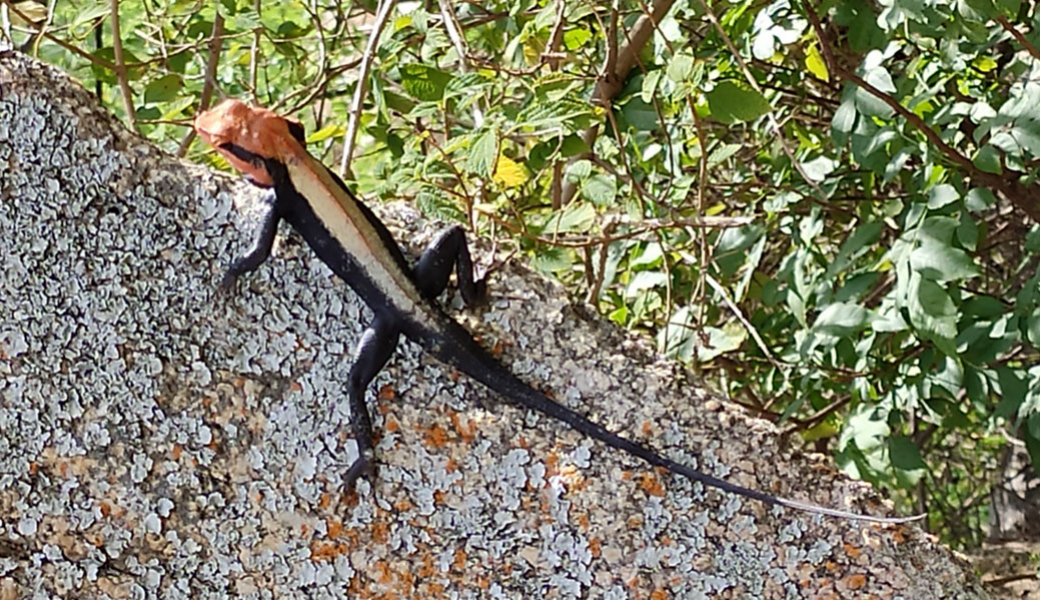
A *P. dorsalis* male in its natural habitat showing mating colours.

**Which part of this research project was the most rewarding?**

I feel it is sometimes risky to work with lizards that involves external stress treatments as the lizards can stop responding and become inactive. I was pleased to see that our lizards remained responsive, which allowed us to successfully complete the experiment on time. Also, I was delighted to present this work at SEB 2025 conference in Antwerp.

**What do you enjoy most about being an early-career researcher?**

What I enjoy the most is the opportunity to ask my own questions and seek the answers. The challenges of failed experiments, time constraints and tight deadlines are unavoidable, but it is always exciting to uncover the results, then shaping them into a story and finally sharing it with all through publications.

**What piece of advice would you give to the next generation of researchers?**

Sometimes things may not work out the way you have planned. That is fine. Do not let it disappoint you, rather be prepared for it and let it motivate you to enquire further. If needed, there is always help available, you just need to ask for it.

**What's next for you?**

Currently, am looking for postdoc opportunities. I would like to continue investigating how animals' foraging and nutritional choices vary across different ecological contexts and ultimately shape their survival and reproductive strategies over the long term.
